# LAMP for Human African Trypanosomiasis: A Comparative Study of Detection Formats

**DOI:** 10.1371/journal.pntd.0000865

**Published:** 2010-11-02

**Authors:** Sally L. Wastling, Kim Picozzi, Abbas S. L. Kakembo, Susan C. Welburn

**Affiliations:** 1 Centre for Infectious Diseases, Division of Pathway Medicine, School of Biomedical Sciences, College of Medicine and Veterinary Medicine, University of Edinburgh, Edinburgh, United Kingdom; 2 Department of National Disease Control, Ministry of Health, Nakasero, Kampala, Uganda; International Centre of Insect Physiology and Ecology, Kenya

## Abstract

Loop-mediated isothermal amplification (LAMP) is at the forefront of the search for innovative diagnostics for human African trypanosomiasis (HAT). Several simple endpoint detection methods have been developed for LAMP and here we compare four of these: (i) visualization of turbidity; (ii) addition of hydroxynaphthol blue before incubation; (iii) addition of calcein with MnCl_2_ before incubation and (iv) addition of Quant-iT PicoGreen after incubation. These four methods were applied to four LAMP assays for the detection of human African trypanosomiasis, including two *Trypanozoon* specific and two *Trypanosoma brucei rhodesiense* specific reactions using DNA extracted from cryo-preserved procyclic form *T. b. rhodesiense*. A multi-observer study was performed to assess inter-observer reliability of two of these methods: hydroxynapthol blue and calcein with MnCl_2_, using DNA prepared from blood samples stored on Whatman FTA cards. Results showed that hydroxynaphthol blue was the best of the compared methods for easy, inexpensive, accurate and reliable interpretation of LAMP assays for HAT. Hydroxynapthol blue generates a violet to sky blue colour change that was easy to see and was consistently interpreted by independent observers. Visible turbidity detection is not possible for all currently available HAT LAMP reactions; Quant-iT PicoGreen is expensive and addition of calcein with MnCl_2_ adversely affects reaction sensitivity and was unpopular with several observers.

## Author Summary

Human African trypanosomiasis (HAT) is a disease of the rural poor in sub-Saharan Africa where diagnostic laboratories are scarce and often ill equipped. Specific LAMP (loop-mediated isothermal amplification) tests for HAT have been developed and represent a significant step forward in the search for simple, sensitive and reliable diagnosis. Easy, accurate and reliable methods to read the results of these tests are critical and several simple methods have been developed. In this study, four methods were compared including three different colour change methods, and one in which the reaction turned from clear to cloudy. The hydroxynaphthol blue method involving a colour change, from violet to sky blue, was easy to see, the test is cheap to use and the results were largely agreed upon by 33 independent observers.

## Introduction

Loop-mediated isothermal amplification (LAMP) [1] is a DNA amplification technique whose advantages over traditional PCR have put it at the forefront of the search for innovative new diagnostics for infectious diseases such as human African trypanosomiasis (HAT) [2]. Rapid and unambiguous visual discrimination of test results is essential for diagnostics and several simple endpoint detection methods have been developed for the LAMP method to allow visual discrimination of positive samples. These methods vary in cost and technological details.

The *Trypanosoma brucei* s.l. species complex includes both agents of HAT namely *Trypanosoma brucei rhodesiense* and *Trypanosoma brucei gambiense*. Two LAMP assays have recently been developed for the detection of all members of the sub genus *Trypanozoon*, which includes *Trypanosoma brucei* s.l. One of the assays targets the single copy paraflagellar rod protein A (*PfrA*) gene [3] and the other the mobile genetic element (RIME) [4]. Further, Thekisoe *et al.* have described a LAMP assay for the detection of *T. b. gambiense* which targets the 5.8S rRNA region [5]. This is controversial since the only widely accepted marker for specific identification of *T. b. gambiense* is the *T. gambiense* specific glycoprotein (*TgsGP*) gene [6], [7]. A *T. b. rhodesiense* specific LAMP assays which targets the serum resistance associated (*SRA*) gene, has also been developed [8]. In addition a second LAMP assay for the *SRA* gene has been developed in our laboratory (Wastling and Picozzi, unpublished observations).

Positive LAMP reactions can be distinguished by visible turbidity in the reaction tube, corresponding to the production of magnesium pyrophosphate, a by-product of DNA amplification [9]. This method was reported to provide immediate visual discrimination of the LAMP output for the *PfrA* assay [3] but neither the RIME nor *SRA*1 assays generate visible turbidity [4], [8]. Quant-iT PicoGreen [10] is a DNA intercalating dye that can be added to the LAMP reaction tube post-incubation. In the presence of LAMP amplification product the dye shows an orange to green colour change and fluoresces under UV light, however the requirement of tube opening to add the dye is a contamination prone step. In contrast calcein, manganese chloride (MnCl_2_), and hydroxynaphthol blue can be added before incubation. Calcein and hydroxynaphthol blue are metal ion indicators, which respond to alterations in the chemical composition of the reaction mix as DNA amplification proceeds. Calcein and MnCl_2_ are reported to produce an orange to green colour change, and to fluoresce under UV light [11], while hydroxynaphthol blue produces a colour change, from violet to sky blue [12].

In the present work four LAMP assays for the detection of human-infective African trypanosomes were compared; the published *PfrA*, RIME and *SRA* assays as a well as a novel LAMP assay for the *SRA* gene developed in our laboratory. Henceforth the published and unpublished LAMP *SRA* assays will be referred to as *SRA1* and *SRA2* respectively. Firstly, purified trypanosome DNA was used to establish the ease of use and sensitivity of each method. Up to this point all endpoint interpretations were made by one observer. Since the performance of a subjective diagnostic method depends on sample variation in readers, as well as cases [13], we performed a multi-observer study to investigate the reliability of the two metal ion indicator methods. These methods were chosen because they are cheap and offer a closed system format.

## Materials and Methods

### LAMP reactions

Four LAMP assays were used. The *PfrA* [3] and RIME LAMP [4] tests specific for the sub-genus *Trypanozoon* and the *SRA* LAMP [8] for *T. b. rhodesiense*, herewith referred to in this publication as *SRA*1 were performed following the published conditions. In addition a novel *SRA* LAMP test (Table 1) targeting *SRA* sequence (GenBank accession number AF097331) was developed and evaluated.

**Table 1:** LAMP primer sequences for the *SRA*2 assay for *T. b. rhodesiense*.

Target gene	Primer	Sequence
*SRA*	FIP	ACGCTATTGGCGCAAGACTTCATAGTGACAAGATGCGTAC
	BIP	ACCAGTGGGCACATCTCAGATATGCACTTTCCTTCTGTCT
	F3	ATCTCAGCGCTTTATGCC
	B3	GCCTTATTGCTACTGTTGTT
	LF	AGCGTGGACTGCGTTGA
	LB	AGTAATCGACATTCTGCAGCAG

The LAMP *PfrA*, RIME and *SRA*1 assays were performed as described [3], [4], [8] except where the reaction buffer detergent was altered between 0.1% Tween 20 and 0.1% Triton X-100. Originally LAMP *PfrA* was run using Tween 20, whereas RIME LAMP, *SRA*1 and *SRA*2 used Triton X-100. For the *SRA*2 LAMP each 25 µl reaction contained 1µl template DNA, 8U *Bst* DNA polymerase (New England Biolabs), 2.5µl Thermopol reaction buffer I (New England Biolabs) with additional MgSO_4_ to give 20 mM Tris-Cl, 10 mM KCl, 10 mM (NH_4_)SO_4_, 8 mM MgSO_4_ and 0.1% Triton X-100, 0.8 M betaine, 1.4 mM dNTPs, 2 µM of both FIP and BIP, 0.2 µM of both F3 and B3 and 0.8 µM of both LF and LB. The reaction was carried out at 62°C for 1 hour, terminated at 80°C for 4 minutes and held indefinitely at 4°C.

### Comparing the four detection formats

The four detection formats were first compared using *T. b. rhodesiense* DNA extracted from cryopreserved procyclic form trypanosomes using a QiaAMP DNA Blood Midi Kit (Qiagen, UK). The concentration of the *T. b. rhodesiense* DNA sample was measured using a NanoDrop spectrophotometer. A 10-fold dilution series was made ranging from a 1 in10 to a 1 in 10, 000, 000 dilution to determine the detection limit for each format-assay combination.

### Turbidity

Turbidity was investigated using the LAMP *PfrA* and LAMP *SRA*2 assays, both of which were performed with Triton X-100 in the reaction buffer (a minor modification to the published LAMP *PfrA* format). Visible turbidity detection is not possible for LAMP RIME or LAMP *SRA*1. Each assay was performed once on the *T. b. rhodesiense* DNA dilution series described above. Post-reaction turbidity was assessed by eye. It was scored as positive or negative in comparison to a positive and negative control. The reaction products were then subjected to gel electrophoresis for approximately 15 min at 100 V using a 1% (w/v) agarose gel containing GelRed (Biotium, UK). The positive LAMP reactions appear as a ladder of bands upon UV illumination. The turbidity and gel electrophoresis results were directly compared.

### Quant-iT PicoGreen

All four assays were performed in duplicate using the *T. b. rhodesiense* DNA dilution series described above with Triton X- 100 in the reaction buffer (a minor modification to the published LAMP *PfrA* format). After the full LAMP reaction incubation time 5 µl of the LAMP product was aliquoted for gel electrophoresis, as above. Then, 2 µl Quant-iT PicoGreen was added to one replicate and 5 µl Quant-iT PicoGreen was added to the second. The colour under indoor light was assessed by eye and recorded and flourescence under UV was also observed, before the colour, fluorescence and gel electrophoresis results were compared.

### Hydroxynaphthol blue

The four assays were performed once on the *T. b. rhodesiense* DNA dilution series described above .with Triton X-100 in the reaction buffer (a minor modification to the published LAMP *PfrA* format). Before incubation 120 µM hydroxynaphthol blue was added to each 25 µl reaction mix. Upon termination the colour was assessed by eye, under indoor light, before the LAMP products were assessed by gel electrophoresis. Colour and gel electrophoresis results were compared.

### Calcein with MnCl_2_

All four assays were performed in triplicate with both reaction buffer compositions (0.1% Tween 20 and 0.1% Triton X-100) using the *T. b. rhodesiense* DNA dilution series described above. Before incubation 25 µM calcein and 0.5 mM MnCl_2_ were added to each 25 µl reaction mix. Upon termination the colour was assessed by eye, fluorescence under UV observed and the LAMP products were assessed by gel electrophoresis. Colour, flourescence and gel electrophoresis results were compared.

### Multi-observer study

Sixty blood samples from human sleeping sickness patients were spotted on to Whatman FTA cards and prepared as described above to obtain DNA eluate. Two 1.2 mm discs were taken from each blood spot and eluted into a final volume of 50 µl chelex solution. The *SRA2* LAMP assay was performed, in duplicate, for each sample. The first reaction included hydroxynaphthol and the second included calcein and MnCl_2_, and Tween- 20 instead of Triton X-100. Five µl of product from each LAMP reaction was electrophoresed through a 1% (w/v) agarose gel, containing GelRed (Biotium, UK) at 100 V. When viewed under UV light, positive LAMP reactions were seen as a long DNA ladder.

Thirty three observers each scored all 60 samples as positive or negative by comparison with a positive and negative control, after training on a small batch of 8 samples. The participants were not chosen according to any specific criteria but were asked about their work background, previous experience of colour change assays and their impressions of these tests in light of their previous experience. They were asked to rank how easy they found it to see the colour difference (very easy, quite easy, quite difficult or very difficult), and how many of the samples were easy to rate as positive or negative (all, most, some, very few or none), for each method. They were also asked which colour change method they found easiest to use. Finally they were given the opportunity to make any general comments.

The results from each observer were compared to the status of each sample, as defined by gel electrophoresis and UV illumination. The agreement between each observer and the gel result was quantified using Cohen's kappa statistic (κ) [14]. The overall level of agreement between all observers was then quantified [15] for both colour change detection methods.

### Ethical statement

Samples were collected as part of ongoing national sleeping sickness surveillance by the Ministry of Health in Uganda. Ethical permission was obtained from Uganda National Council for Science and Technology (UNCST) as well as the District Health Authorities of Uganda.

## Results

### Turbidity

Positive LAMP *PfrA* reactions were detectable under visible turbidity up to and including a 1×10^−4^ dilution of the *T. b. rhodesiense* DNA. This was equal to the detection limit seen when the same reaction products were visualised by UV illumination after gel electrophoresis. Similarly, positive LAMP *SRA*2 reactions were detectable by visible turbidity up to and including a 1×10^−3^ dilution of the *T. b. rhodesiense* DNA. This was equal to the detection limit seen when the same reaction products were visualised by UV illumination after gel electrophoresis. Therefore the same results were seen with turbidity and gel electrophoresis for these assays. However visible turbidity detection is not possible for LAMP RIME or *SRA*1. These results are shown alongside the detection limits with other endpoint readout formats in Table 2.

**Table 2:** Detection limit with different assays and detection methods with a 10 fold dilution series of 13.6 nM *T. b. rhodesiense* DNA.

		*PfrA*	RIME	*SRA1*	*SRA2*
**Turbidity**	Turbidity	1×10^−4^	Not done	Not done	1×10^−3^
	Gel	1×10^−4^	Not done	Not done	1×10^−3^
**Quant-iT PicoGreen (2 µl)**	Colour	1×10^−3^	1×10^−5^	1×10^−4^	1×10^−3^
	Fluorescence	1×10^−3^	1×10^−5^	1×10^−4^	1×10^−3^
	Gel	1×10^−4^	1×10^−5^	1×10^−4^	1×10^−3^
**Quant-iT PicoGreen (5 µl)**	Colour	1×10^−4^	1×10^−5^	1×10^−4^	1×10^−4^
	Fluorescence	1×10^−4^	1×10^−5^	1×10^−4^	1×10^−4^
	Gel	1×10^−4^	1×10^−5^	1×10^−4^	1×10^−4^
**Hydroxynaphthol blue**	Colour	1×10^−5^	1×10^−5^	1×10^−3^	1×10^−3^
	Gel	1×10^−5^	1×10^−5^	1×10^−3^	1×10^−4^

### Quant-iT PicoGreen

**Figure 1:**
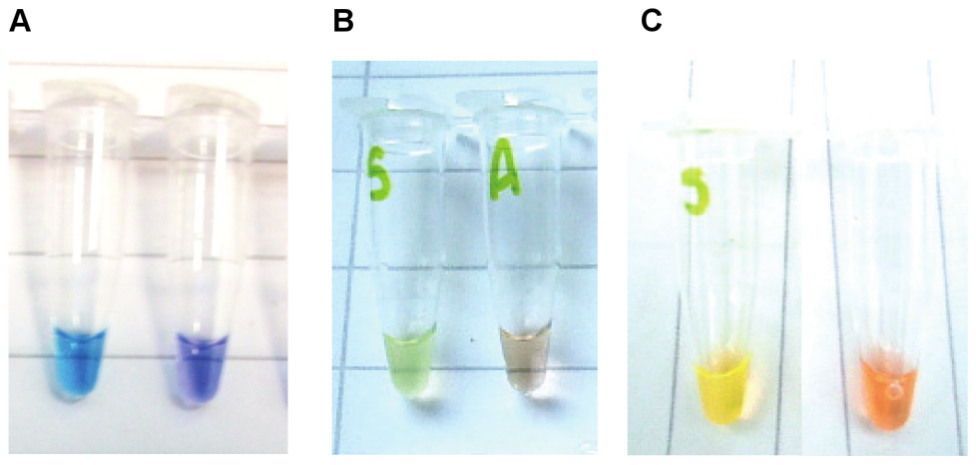
Examples of colour changes seen with hydroxynaphthol blue, calcein with MnCl_2_ and Quant-iT PicoGreen. Panel A shows the colour change seen with hydroxynaphthol blue, panel B shows the colour change seen with calcein and MnCl_2_ and panel C shows the colour change seen with Quant-iT PicoGreen.

Upon the addition of either 2 or 5 µl of Quant-iT PicoGreen to the LAMP reaction products positive reactions could be detected by an orange to green colour change visible under normal light, or by fluorescence under UV light. This was true for each assay, whose detection limits with these formats are shown in Table 2. For LAMP RIME, *SRA*1 and *SRA*2 colour and fluorescence based detection showed perfect agreement with gel electrophoresis regardless of the volume of Quant-iT PicoGreen. For LAMP *PfrA* with 2 µl Quant-iT PicoGreen colour and fluorescence based detection disagreed with results by gel in two instances. First, with the 1×10^−4^ dilution gel positive but colour change and flourescence negative endpoints were seen. Second, with the 1×10^−5^ dilution gel negative but colour change and fluorescence positive endpoints were seen. With 5 µl of this reagent colour change and fluorescence based detection agreed with gel electrophoresis for all dilutions. These results reflect the author's personal feeling that colour and fluorescence were more easily discerned with 5 µl, rather than 2 µl of Quant-iT PicoGreen. An example of the colour change seen with Quant-iT PicoGreen can be seen in Figure 1.

### Hydroxynaphthol blue

Hydroxynaphthol blue also allowed positive reactions to be discerned under ambient light for all four LAMP assays (Table 2). Hydroxynaphthol blue induced colour change showed perfect agreement with detection by gel for all assays except *SRA*2 when one gel positive assay (corresponding to the 1×10^−4^ dilution) appeared violet (negative). No false positives were seen with hydroxynaphthol blue versus the gel. Also, for the LAMP *PfrA*, RIME and *SRA2* assays, inclusion of hydroxynaphthol blue had no effect on overall detection limit. However the sensitivity of the LAMP *SRA1* was reduced when hydroxynapthol blue was added compared to those results seen with Quant-iT PicoGreen (Table 2). An example of the colour change seen with hydroxynaphthol blue can be seen in Figure 1.

### Calcein and MnCl_2_

The calcein and MnCl_2_ method was found to give results of variable quality. No positive LAMP amplification was seen with the RIME assay, with either reaction buffer, despite running each assay in triplicate for the full dilution series. Neither were any LAMP positive endpoints seen for the LAMP *PfrA* or LAMP *SRA1* reactions with 0.1% Triton X-100 containing LAMP buffer, despite running each assay in triplicate for the full dilution series. Amplification was seen when 0.1% Tween 20 was used. For LAMP *PfrA* with 0.1% Tween 20 consistent amplification could be seen from the 1×10^−2^ dilution when the products were assessed by gel, but the colour change and fluorescence were ambiguous. For LAMP *SRA*1 two replicates were performed with 0.1% Tween 20, one showed no amplification, and one amplified from 1×10^−1^, 1×10^−2^, 1×10^−3^ and 1×10^−5^ dilutions when the products were assessed by gel. Colour change and flouresence were seen for the first three of these amplifications. For LAMP *SRA2* amplification was seen with both types of buffer, although the detection limit was variable across the three replicates for each buffer. For one replicate colour change and fluorescence were visible and agreed with the results by gel. For the second replicate 2/4 and 3/4 gel positive endpoints were seen by colour change and fluorescence respectively. For the third replicate there was no obvious difference between gel positive and negative reactions by colour or fluorescence. Furthermore, the inclusion of calcein and MnCl_2_ seemed to reduce the absolute sensitivity of the assay as compared to results seen with turbidity or Quant-iT Picogreen, where extra reagents were not added to the reaction mix. Consistent amplification from the 1×10^−3^ dilution was seen by turbidity, Quant-iT PicoGreen and hydroxynapthol blue. With calcein and MnCl_2_ this was reduced to 1×10^−1^. An example of the colour change seen with Quant-iT PicoGreen can be seen in Figure 1.

### Participants in the multi-observer study

Thirty three volunteers participated in this study. There was a strong occupational bias towards the biomedical sciences. Eighteen participants self defined as biological scientists, six were veterinarians (of which, three also described themselves as biologists) and one was a medical doctor. Nine participants did not place themselves into any of the above categories. Nine participants reported some previous experience with diagnostic tests, which require a colour or colour change to be observed, although in most cases this experience was limited. Twenty one of the participants were female, twelve were male. Age was not surveyed.

### Questionnaire

All observers said that they found the violet to blue colour change, seen with hydroxynaphthol blue, easier to use than the orange to green colour change seen with calcein and MnCl_2_.

When asked, ‘In your opinion, how easy is it to see the violet to sky blue colour/orange to green difference?’, 73% of observers found the colour change with hydroxynaphthol blue quite easy to see, whereas 94% of the observers found the colour change with calcein and MnCl_2_ very difficult to see. When asked ‘In your opinion, using this colour change, how many of the samples were easy to rate as positive or negative?’ all participants found most or some of the samples easy to score with hydroxynaphthol blue. Equally all participants found very few or no samples easy to score with calcein and MnCl_2_.

Of those who had some previous experience interpreting diagnostic tests both methods were generally considered to be less easy to interpret than their previous experiences.

Eight observers commented on a difference in turbidity between the positive and negative controls for the calcein and MnCl_2_ method. Two participants found it easier to score the samples using this turbidity difference than with the colour difference seen with hydroxynaphthol blue. The participants were not closely questioned as to how they made their decision; it is possible that several more made their judgements on a similar basis.

### Individual observer agreement with reference standard method

Cohen's kappa statistic (κ) [14] was used to compare the agreement of observers with the results according to gel electrophoresis. For hydroxynaphthol blue agreement ranged from κ = 0.145 to κ = 0.870, averaging κ = 0.602, and for calcein and MnCl_2_ agreement ranged from κ<0 to κ = 0.930, averaging κ = 0.407. For 66% of observers the agreement between the hydroxynaphthol blue colour change and the results seen by gel electrophoresis were better than agreement between the calcein MnCl_2_ colour change and gel.

### Inter-observer agreement

The method of Fleiss, Levin et al. [15] was used to quantify the agreement of all observers for each method. For hydroxynaphthol blue, a kappa value of 0.693 was significantly different from zero - or no agreement, (P<0.0001). For calcein and MnCl_2_ a kappa value of 0.209 was seen. This was also significantly different from zero (P<0.0001). It is clear that the agreement between many observers was much higher for the hydroxynapthol blue method.

## Discussion

LAMP is advocated as a low technology diagnostic tool for resource poor settings [2]; simple visual discrimination of the test result is perceived as an important factor in promoting the method as a straightforward diagnostic [12]. However LAMP results can be read in a variety of ways. Complex sequence specific [16] and high technology, real time turbidimetry [17] approaches are useful during assay design and optimisation. LAMP products may also be visualised directly following gel electrophoresis by UV transillumination. More simply, turbidity is generated as a by-product of DNA amplification [9]. Several colour change methods for reading the result within the reaction tube have also been developed, including the DNA intercalating dyes: Quant-iT PicoGreen [10], SYBR Green I [18], [19] and propidium iodide [19] and the metal ion indicator methods: calcein alone [20], calcein with MnCl_2_ [11] and, most recently, hydroxynaphthol blue [12]. The metal ion indicators have provided the simplest approach to date; they are added alongside the other reagents, before incubation, so that amplification and detection are combined in single processing step, within a closed tube system. The colour changes can be visualised by eye, without special lighting, and they are inexpensive.

One important caveat must be acknowledged; LAMP endpoint detection is not sequence specific whether by gel electrophoresis, turbidity or colour change dyes. Unlike PCR it is not possible to deduce the identity of the amplicon by examining the ladder-like pattern on the gel. Rather, once a given LAMP assay has been developed it is assumed that all reaction products correspond to the intended target. Amplification specificity is considered extremely high because LAMP primers must bind six distinct regions on the target DNA [1], [12].

Turbidity enabled sensitive and specific endpoint discrimination for LAMP *PfrA* and *SRA*2 but is not visible for the LAMP RIME or *SRA1* assays using published protocols. Quant-iT PicoGreen enabled easy visual endpoint discrimination for all assays. It was easiest to see the colour change with 5 µl (rather than 2 µl ) of this reagent, but the extra cost is significant for this expensive reagent. Simple, sensitive and specific endpoint discrimination was also possible for all assays using the hydroxynaphthol blue method. For LAMP RIME, *PfrA* and *SRA*2 inclusion of hydroxynapthol blue did not affect the detection limit of the assay, confirming previous work [12] that this reagent does not inhibit the LAMP reaction. However, a ten fold reduction in detection limit was seen for the LAMP *SRA1* assay with hydroxynapthol blue compared to assays without any additional reagents in the reaction mix. Further replicates would need to be made to confirm this observation. Goto *et al.* [12] also reported Mn^2+^ inhibition of LAMP, which may explain the total inhibition of LAMP RIME, and partial inhibition of LAMP *PfrA*, *SRA1* and *SRA2* assays found in the present work when calcein and Mn^2+^ was added.

This work demonstrates that each detection method should be validated for any given LAMP assay. This is particularly important for the turbidity and metal ion indicator approaches, which detect changes in the chemical composition of the reaction mix rather than amplified DNA. Hydroxynaphthol blue was found to be the better of the two metal ion indicator methods tested, while the calcein and MnCl_2_ method reduced LAMP reaction sensitivity. Even so, the reliability of hydroxynaphthol blue should be confirmed across a larger set of independent observers.

Inter-reader variability is an issue that has been largely ignored in the LAMP literature to date, and must be addressed where subjective endpoints are advocated as useful tools. In previous work endpoint interpretations have been made by one observer and to our knowledge no large scale, multi-observer studies have been performed with any of the LAMP endpoint detection methods. As Sadatsafavi *et al.* have emphasised, ‘any attempt to generalize the performance of a subjective diagnostic method should take into account the sample variation in both cases and readers’ [13]; they further highlight the need for a large group of observers to be used. We have performed a multi- observer study with 33 participants to investigate the reliability of the two metal ion indicator methods used in LAMP diagnostics. These methods were chosen for their advantages as closed system methods and their low cost. By contrast Quant-iT PicoGreen is expensive, and breaks the closed system. Turbidity was excluded based on the author's perception that it required a ‘trained eye’ to detect, although this may be a false assumption, and because it is not possible with all of the currently available HAT LAMP assays.

The agreement across all observers was better for hydroxynaphthol blue tests (κ = 0.693) than for calcein with MnCl_2_ (κ = 0.209), and, for 66% of observers the hydroxynaphthol blue to gel agreement was better than calcein MnCl_2_ to gel. All observers said they found the hydroxynaphthol blue colour change easier to see. Several observers commented on a turbidity difference between positives and negatives for the calcein-MnCl_2_ method. Six of the 11 observers whose calcein-MnCl_2_ to gel agreement was better than that for hydroxynaphthol blue commented on this turbidity difference. Thus the orange to green colour change may be even less reliable than this report suggests.

The present work has shown that hydroxynaphthol blue is the better of the two metal ion indicator methods tested. Not only is it easier to see, but it also shows better inter- reader reliability and more consistent agreement with the presence of the DNA amplicon, assessed by gel. However, Cohen's kappa statistic for the agreement between the colour change as interpreted by one observer and amplicon detection by electrophoresis with UV illumination, ranged from κ = 0.145 to κ = 0.870. Therefore the agreement between hydroxynaphthol blue and gel electrophoresis is variable and imperfect across many observers. We conclude that hydroxynaphthol blue could be well suited as a tool to increase the efficiency of large scale screening and monitoring efforts but when more than one individual is involved in LAMP screening, training and quality control will be necessary to reduce inter-observer variation. This process might be aided by generating a colour swatch card against which assay results can be compared, along the lines of a litmus paper test for pH. Better still, a quantitative colour measure, using some kind of spectrophotometric device would remove this variability, although we must not forget that we are seeking to establish a LAMP endpoint detection format that is suitable in a low resource setting.

This work also provided a snapshot on the running costs for each fo the four LAMP detection formats. The Quant-iT PicoGreen format was the most expensive of the methods used with a cost of $353 for 100 reactions. By sharp contrast the use of turbidity, hydroxynaphthol blue and calcein with MnCl_2_ were less than $0.01 for 100 reactions (Table 3.). Cost is a major factor in endemic areas. As such hydroxynaphthol blue seems the most prefereable method when cost and sensitivity are considered together.

**Table 3:** Costs associated with the methods investigated in this study, per 100 reactions (prices are based on UK reagent prices at the time of the study, and are converted to US $).

Method of endpoint detection	Cost per 100 reactions
Quant-iT PicoGreen	US $ 353.06
Turbidity	US $ 0.00
Hydroxynaphthol blue	US $ 0.0009
Calcein and MnCl_2_	US $ 0.0011

In conclusion, hydroxynaphthol blue was the best method for easy, inexpensive, accurate and reliable interpretation of LAMP assays for human African trypanosomiasis. The violet to sky blue colour change was easy to see and was more consistently interpreted by independent observers. A range of problems were seen with the other methods. Visible turbidity is not possible for all LAMP HAT assays. Quant-iT PicoGreen performed excellently, but opening the reaction tube exposes the laboratory to product contamination. It is also at least 15 times more expensive than the other methods. With calcein and MnCl_2_ the four assays showed a range of partial to total inhibition and the colour change was difficult to see leading to poor agreement between several independent observers.

However, hydroxynaphthol blue is not perfect. We have shown that the agreement between amplicon detection by gel electrophoresis and UV illumination and colour change by hydroxynaphthol blue can be variable and imperfect for different observers. Therefore, while hydroxynaphthol blue is a promising method for low technology LAMP endpoint detection, further work is required to develop methods that will assist different observers to make consistent interpretations of the same colour change.
